# Exploring the distinctive characteristics of gut microbiota across different horse breeds and ages using metataxonomics

**DOI:** 10.3389/fcimb.2025.1590839

**Published:** 2025-07-07

**Authors:** Xinxi Qin, Li Xi, Longfei Zhao, Jincheng Han, Hongxia Qu, Yajun Xu, Weiping Weng

**Affiliations:** ^1^ College of Biology and Food, Shangqiu Normal University, Shangqiu, China; ^2^ Department of Research and Development, Inner Mongolia Huatian Pharmaceutical Co., Ltd, Chifeng, China

**Keywords:** 16S rRNA, breed, age, microbiota, horse

## Abstract

**Background:**

Gut microbiota exerts a pivotal function in host nutrient metabolism and maturation of the mucosal immunity. Analyzing the reciprocal interaction between horses and gut microbiota constitutes a crucial aspect of scientific feeding practices. This study aims to investigate the differences in gut microbiota among Hequ horses, Mongolian horses, and Thoroughbred horses, as well as between Thoroughbred horses at two age stages.

**Methods and results:**

Paired-end sequencing with a read length of 2×250 bp targeting the V3-V4 region of the 16S rRNA gene in fecal samples was carried out. Subsequently, differences in the diversity, composition, and metabolic pathways of the gut microbiota among the groups were analyzed. The results showed that: (1) Horse breeds were associated with variations in the gut microbiota. Microbial diversity, the proportion of commensal bacteria from Bacillota and Bacteroidota, and bacterial communities involved in dietary fiber metabolisms were significantly higher in the gut of the Hequ horses than in the gut of the Mongolian and Thoroughbred horses. The highest Bacillota to Bacteroidota (B/B) ratio and enrichment of bacterial communities involved in the metabolism of bile acids, lipids, and amino acids in the gut of the Mongolian horses resulted in significantly higher lipid metabolism and amino acid metabolism than in the other two breeds. The bacterial communities enriched in the gut of Thoroughbred horses were primarily involved in carbohydrate metabolism, but the level of energy metabolism was significantly lower than in Hequ horses. (2) The results also showed an association between age and gut microbiota of Thoroughbred horses. The alpha diversity, B/B ratio, and 83.33% of metabolic pathways did not differ significantly between younger and older Thoroughbred horses. However, there were significant differences between the two age groups in beta diversity, composition of glycolytic bacteria, metabolism of cofactors and vitamins, and energy metabolism of gut microbiota.

**Conclusions:**

Collectively, these results point to an association between the breed of horses or the age of Thoroughbred horses with variations in gut microbiota. The current findings will serve as a reference for improving feeding strategies for horses.

## Introduction

The gut microbiota assumes a pivotal role in regulating the development of the host’s gastrointestinal tract and facilitating the processes of digestion and absorption. For monogastric herbivores, a rich and diverse gut microbiota is essential for upholding intestinal health and fermenting fibers (Collinet et al., 2021; Li et al., 2022a). Beneficial flora, on one hand, can prevent pathogen invasion by adhering to the intestinal mucosal epithelium (Ericsson et al., 2016). Moreover, Zhou et al. (2022) discovered that the gut of wild horses had a higher abundance of Christensenellaceae and Oscillospiraceae compared to captive horses. These two bacterial families were significantly negatively correlated with intestinal metabolic disorders and were involved in the anti-inflammatory process (Zhou et al., 2022). Research has indicated that alterations in feeding patterns can cause rapid changes in the structure of the gut microbial community, thereby increasing the risk of abdominal colic (Lara et al., 2022). On the other hand, hindgut microorganisms are capable of fermenting plant fibers, such as cellulose, hemicellulose, starch, and pectin, to produce short-chain fatty acids (SCFAs). These SCFAs supply 60-70% of the energy requirements for horses (Vermorel and Martin-Rosset, 1997). The majority of fiber-degrading bacterial communities in the herbivore gut, like those from the Lachnospiraceae and Ruminococcaceae, belong to the phylum Bacillota (Garber et al., 2020). In addition, Prevotellaceae and Rikenellaceae from the phylum Bacteroidota, and Fibrobacteraceae from the phylum Fibrobacteres also have polysaccharides fermentation capacity (Gharechahi et al., 2020; Su et al., 2020; Weinert-Nelson et al., 2022).

The composition and relative stability of gut microbiota are closely associated with the health of horses. However, numerous factors can impact the horse gut microbiota, including breed, age, physiological status, weaning, diet type, intestinal diseases, and the feeding environment (Garber et al., 2020; Arnold et al., 2021). Several studies have confirmed an apparent association between breed and gut microbiota of horses. For instance, Hernández-Quiroz et al. (2022) found differences in the dominant gut microbiota among seven Mexican horse breeds (Hernández-Quiroz et al., 2022). Massacci et al. (2020) conducted a comparison of the gut microbiota of six horse breeds. Their research revealed that 27 bacterial genera differed significantly between breeds, and nine of these genera were confirmed to be heritable in humans (Massacci et al., 2020). In two separate studies that compared the gut microbiota of Thoroughbred horses with those of two other breeds (Jeju horses in Korea and Mongolian horses in China), Thoroughbred horses exhibited both higher microbial diversity and a greater abundance of SCFAs-producing bacteria (Park et al., 2021; Wen et al., 2022). The microbial community also undergoes adaptive alterations with advancing age. Costa et al. (2016) observed that as foals aged from 0–9 months, the composition of their gut flora changed drastically, accompanied by a decrease in diversity (Costa et al., 2016). The same tendency was noted in two additional studies: one comparing the gut microbiota in horses between the ages of 5-31, and the other between mature horses (ages 5-12) and elderly horses (ages 19-28) (Dougal et al., 2014; Theelen et al., 2021).

The Hequ horses and the Mongolian horses represent two principal horse breeds in China, with their primary production regions located in Qinghai Province and Inner Mongolia Autonomous Region respectively. Both breeds of horses have been listed in National Protected Breeds of Livestock and Poultry Genetic Resources (Yan et al., 2022; Zhang et al., 2025). These horses have the characteristics of good stamina, adaptability to the natural environment, cold resistance, roughage resistance, and disease resistance (Bao et al., 2020). Thoroughbred horses originated in the United Kingdom during the 17th to 18th centuries (Bailey et al., 2024). Currently, the main production areas are mainly in the United Kingdom, the United States, Australia, Japan, and New Zealand. Thoroughbred horses are famous for their fast speed in short and medium distances (McGivney et al., 2020). Additionally, this breed has high genetic stability, extensive adaptability and high breeding value, which effectively improves the performance of other breeds, especially enhancing the high-speed force (Chu et al., 2012; Kim et al., 2018). Since 1950, China has introduced Thoroughbred horses for the first time to improve local breeds. In 2020, Thoroughbred horses were listed as introduced breeds in the National Catalog of Livestock and Poultry Genetic Resources. In captivity, the health and competitive ability of the horse depends largely on scientific rearing. Management of diet using gut microbiota information may improve the corresponding performance of horses. Holo-omics studies found that Lachnospiraceae in the gut of horses down-regulate mitochondrial genes involved in energy metabolism, biogenesis, and Ca^2+^ cytosolic transport, thus reducing the endurance performance of horses (Mach et al., 2022). The study speculated that increased pectin and protein in the diet could mitigate the adverse effects of Lachnospiraceae on horse endurance. Adams et al. (2022) found that the addition of formulated prebiotics to the diet of Thoroughbred racehorses reduced the abundance of Bacteroidota, increased the abundance of Bacillota and Bacillota to Bacteroidota (B/B) ratio (Adams et al., 2022). Metagenomic analysis of the gut microbiome of 242 horses observed 36 metagenomic assembled genomes (MAG) enriched in racehorses, where CAZy gene abundance was significantly correlated with the gender and breed of horses. These acetate-and butyrate-producing microorganisms are expected to be used as biomarkers to identify endurance of racing horses and may be developed as probiotics to promote athletic performance and health of horses (Li et al., 2023). We hypothesize that the breed and age of horses are related to the diversity, composition, and metabolism of the gut microbiota involved.

The present study was designed to investigate the association of breed and age with gut microbiota and to explore the potential association of characteristic microbiota with metabolism in the gut of horses. The experimental data and conclusions will serve as a reference for promoting intestinal health and precision feeding strategies in horses. Moreover, they will facilitate in-depth investigations into the mechanisms underlying the interactions among gut microbiota, nutritional requirements, and production performance in equine species.

## Materials and methods

### Sample collection

The Hequ horses (HQ: 4-year-old stallion, n=5), Mongolian horses (MON: 4-year-old stallion, n=5), and Thoroughbred horses (TBy: 4-year-old stallion, n=5; TBo: 15-year-old stallion, n=5) involved in the trial were supplied by the Fenghe racecourse in Linyi, China. The horses were acquired for the racecourse at the age of 1–2 years. Mongolian and Thoroughbred horses were purchased from Inner Mongolia Rider Horse Industry Ltd., where the Mongolian horses were bred in-house by the company, while the Thoroughbred horses were imported from New Zealand. Hequ horses were sourced from Qinghai Jiuzhi Ecological Animal Husbandry Ltd., which specializes in breeding this particular breed. Each horse was stabled in an individual stall and fed four times daily at 8:00, 14:00, 19:00, and 23:00, with unrestricted access to drinking water. The diet included hay (a combination of oat hay and alfalfa hay) and concentrate. The concentrate was composed of 48.0% corn, 29.0% wheat bran, 17.5% soybean meal, 1.5% vitamin and mineral premixes, 2.4% CaHPO_4_, and 1.6% NaCl. Additionally, the horses underwent regular training, three times a week, covering a distance of six kilometers each time. When feces was defecated by a horse, approximately 5 g of fecal sample immediately collected from the inner portion of the feces using sterile forceps. The sample was then placed into a sterile self-sealing bag, stored in dry ice, transported to the laboratory within 2 h, and stored in a -80°C refrigerator for further study.

### 16S rRNA gene V3-V4 region amplification and sequencing

A 0.5 g fecal sample was weighed, and total bacterial DNA was extracted using the Omega M5635–02 kit (Omega Biotek Inc., USA). The amount and quality of the extracted DNA were determined by the NC2000 UV spectrophotometer (Thermo Scientific, USA) and 1.0% gel electrophoresis. For PCR amplification, specific primers for the V3-V4 region of 16S rRNA gene (338F: 5’-ACTCCTAC GGGAGGCAGCA-3’ and 806R: 5’-GGACTACHVGGGTWTCTAAT-3’) with barcode were synthesized. The amplification was carried out using the Q5 high-fidelity DNA polymerase reaction system (New England Biolabs, USA) under the following reaction conditions: 98°C for 2 min; 98°C for 15 s, 55°C for 30 s, 72°C for 30 s (30 cycles); 72°C, 5 min. The amplified products were recovered and their fluorescence intensities were quantified using the FLx800 Microplate reader (BioTek, USA). The amplified sequence libraries were fabricated with the TruSeq^®^ Nano DNA kit (Illumina, USA), followed by high-throughput sequencing of the clone libraries using the Novaseq 6000 platform in paired-end (2×250 bp) mode (Illumina, USA).

### Bioinformatics analysis

The raw data were initially filtered using Trimmomatic v0.33 software (Bolger et al., 2014). The parameters were set as follows: a window size of 50 bp and an average Q-score threshold of 20. Primer sequences were identified and eliminated with Cutadapt v1.9.1 software (Martin, 2011). The parameters included a maximum accepted mismatch of 20% and a minimum coverage of 80%. The quality controlled data was denoised using the DADA2 v1.20 workflow (Callahan et al., 2016) within QIIME2 v2020.6 software (Bolyen et al., 2019). First, the “filterAndTrim” function was employed to remove low-quality reads, with parameters set as maxN=0, truncQ=2, and maxEE=1. Subsequently, chimeras in the sequences were removed using the “removeChimeraDenovo” function with default parameters. The filtering threshold for amplicon sequence variants (ASVs) was set at 0.005% of the total number of sequences. Species annotation was carried out using the “classify-sklearn” function in QIIME2 software, which is based on the Naive Bayesian classifier. A confidence threshold of 0.7 was set. The Silva database (Release138, https://www.arb-silva.de/) was searched to acquire the taxonomic information of the ASV representative sequences (Quast et al., 2013). The generated ASV table was rarefied by the minimum number of reads for subsequent analysis. The community composition of each group was analyzed at every taxonomic level. The rarefaction curve was used to verify whether the sequencing data volume was adequate to represent the species diversity in the sample. The rank-abundance curve was applied to measure the richness and evenness of the species within the sample. To explore the function of microbial community in the horses gut, the metagenomic functional composition of the 16S rRNA amplicon database was predicted using PICRUSt2 software (Douglas et al., 2020). Subsequently, the differences in the metabolic pathway of the gut microbiota functional genes between the groups were evaluated by the Kyoto Encyclopedia of Genes and Genomes (KEGG, https://www.kegg.jp/) (Altermann and Klaenhammer, 2005) pathway enrichment analysis.

### Statistical analysis

After rarefying all samples to the lowest number of reads among the samples, the Vegan package in R software v3.6.1 was employed to compute both alpha and beta diversity. To examine the disparities in alpha diversity (ACE and Simpson index) and species composition between groups, the Kruskal Wallis rank-sum test was utilized for the comparison among breed groups, while the Wilcoxon rank-sum test was applied for the comparison between age groups. Subsequently, the Benjamini-Hochberg method was used to correct the *P*-values. The distance matrix among samples was generated based on the weighted unifrac distance algorithm (Lozupone and Knight, 2005). Subsequently, a principal coordinates analysis (PCoA) (Gower, 1996) plot was created using the R software v3.6.1. The Linear discriminant analysis (LDA) effect size (LEfSe) analysis (Segata et al., 2011) was used to identify species with significant differences between breeds or ages (LDA threshold: 4.0). The evolutionary distributions of the biomarkers were visualized in the LEfSe cladogram. Differences in KEGG pathways between groups were analyzed using Statistical Analysis of Metagenomic Profiles (STAMP) v2.1.3 (Parks and Beiko, 2010).

## Results

### Sequences analysis

**Figure 1:**
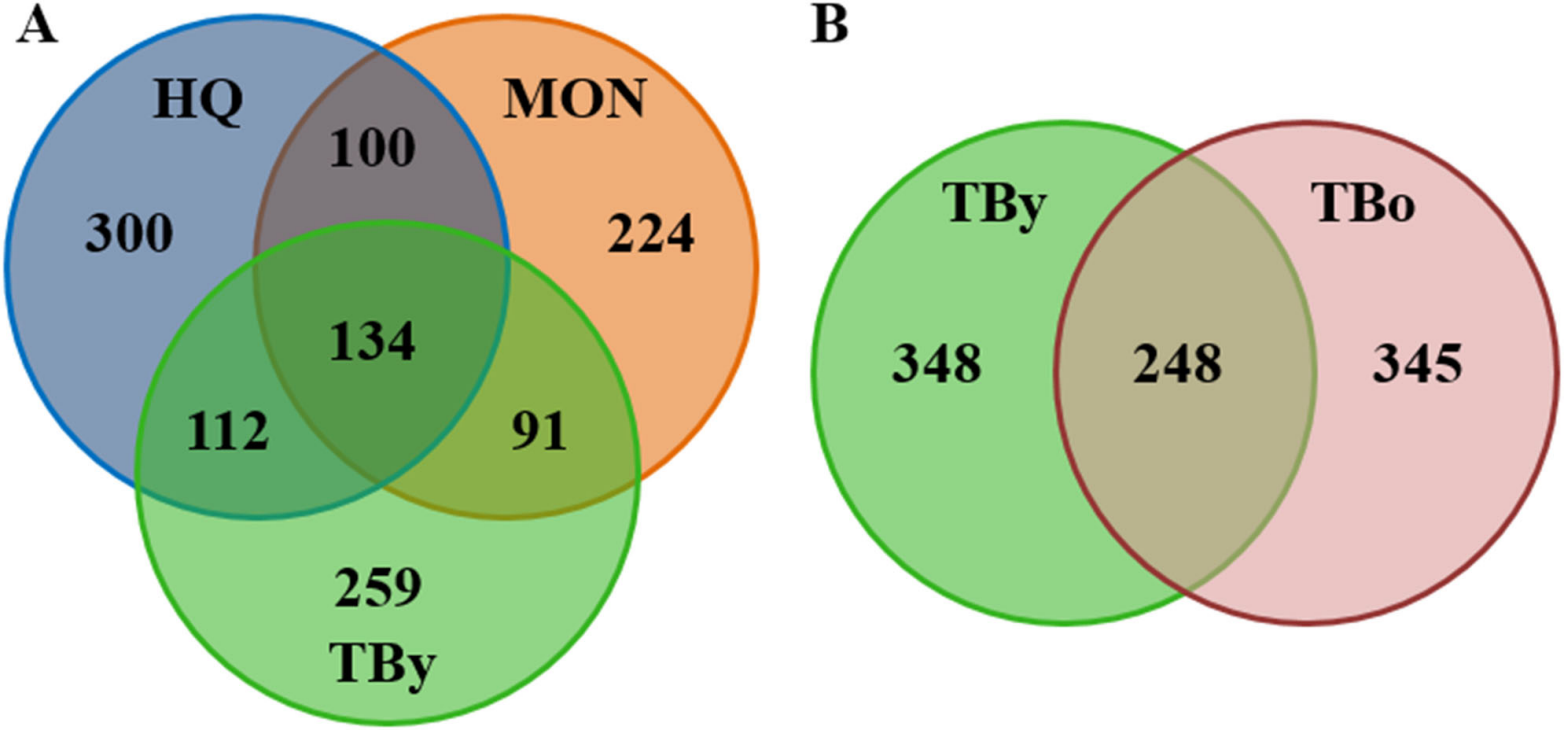
Venn diagrams. **(A)** Distribution diagram of ASVs among the breed groups. **(B)** Distribution diagram of ASVs between the age groups. TBy, The younger Thoroughbred horses; TBo, The older Thoroughbred horses; HQ, Hequ horses; MON, Mongolian horses.

A total of 1,200,800 pair-end reads were generated from 15 samples across three breeds, with a minimum of 79,689 reads per sample. The ASV table was rarefied to 79,689 reads per sample for standardization. Additionally, 800,605 pair-end reads were obtained from 10 fecal samples across two age groups, with a minimum of 79,748 reads per sample, and the corresponding ASV table was rarefied to 79,748 reads per sample. The raw data processing results are presented in **Supplementary Table S1**. Among the three breeds, 1,220 ASVs were clustered in total, and for the two age groups, 941 ASVs were clustered. As depicted in the Venn diagram (**Figure 1A**), the three breeds shared 10.98% of ASVs, and the proportion of unique ASVs was 24.59% for the HQ group, 18.36% for the MON group, and 21.23% for the TBy group. For the Thoroughbred horses, the younger (TBy) and older (TBo) groups shared 26.35% of ASVs, and the proportion of unique ASVs was 36.98% for the TBy group and 36.66% for the TBo group (**Figure 1B**). The rarefaction curves for each sample reached a plateau at the end, suggesting that the sequencing depth of this experiment was adequate (**Supplementary Figures S1**, **S2**). The Rank abundance curves demonstrated that the HQ group exhibited the highest richness and evenness (**Supplementary Figure S3**). Moreover, the TBo group had higher microbiota richness than TBy group (**Supplementary Figure S4**).

### The alpha diversity analysis of the bacterial community

**Figure 2:**
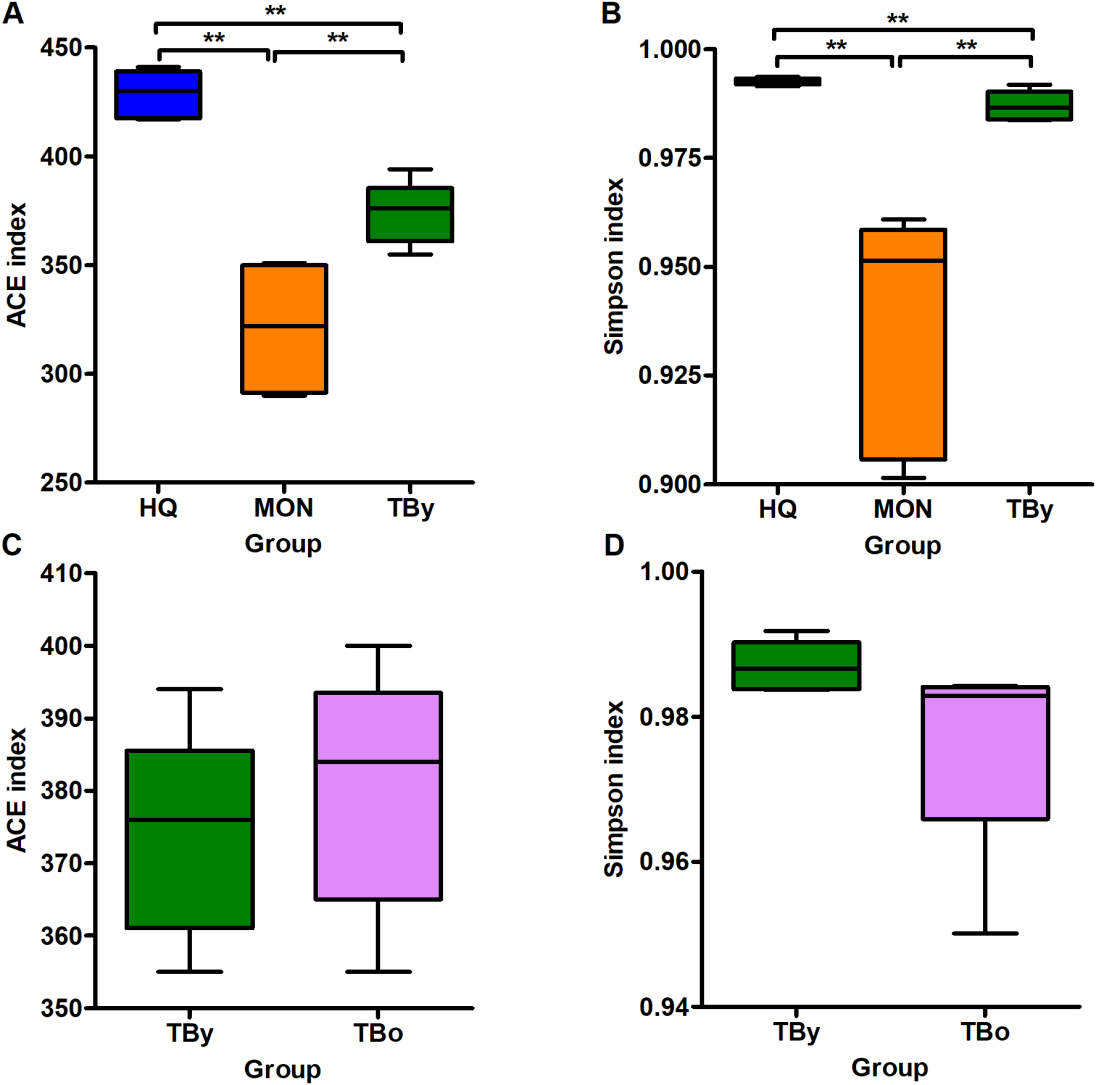
Differences in alpha diversity between groups. **(A)** Comparison of the ACE indices among the breed groups. **(B)** Comparison of the Simpson indices among the breed groups. **(C)** Comparison of the ACE indices between the age groups. **(D)** Comparison of the Simpson indices between the age groups. TBy, The younger Thoroughbred horses; TBo, The older Thoroughbred horses; HQ, Hequ horses; MON, Mongolian horses. Statistic analysis: Kruskal Wallis rank-sum test for the comparison among the breed groups, Wilcoxon rank-sum test for the comparison between the age groups, and *P*-values were corrected using the Benjamini-Hochberg method. **P<0.01.

**Figure 3:**
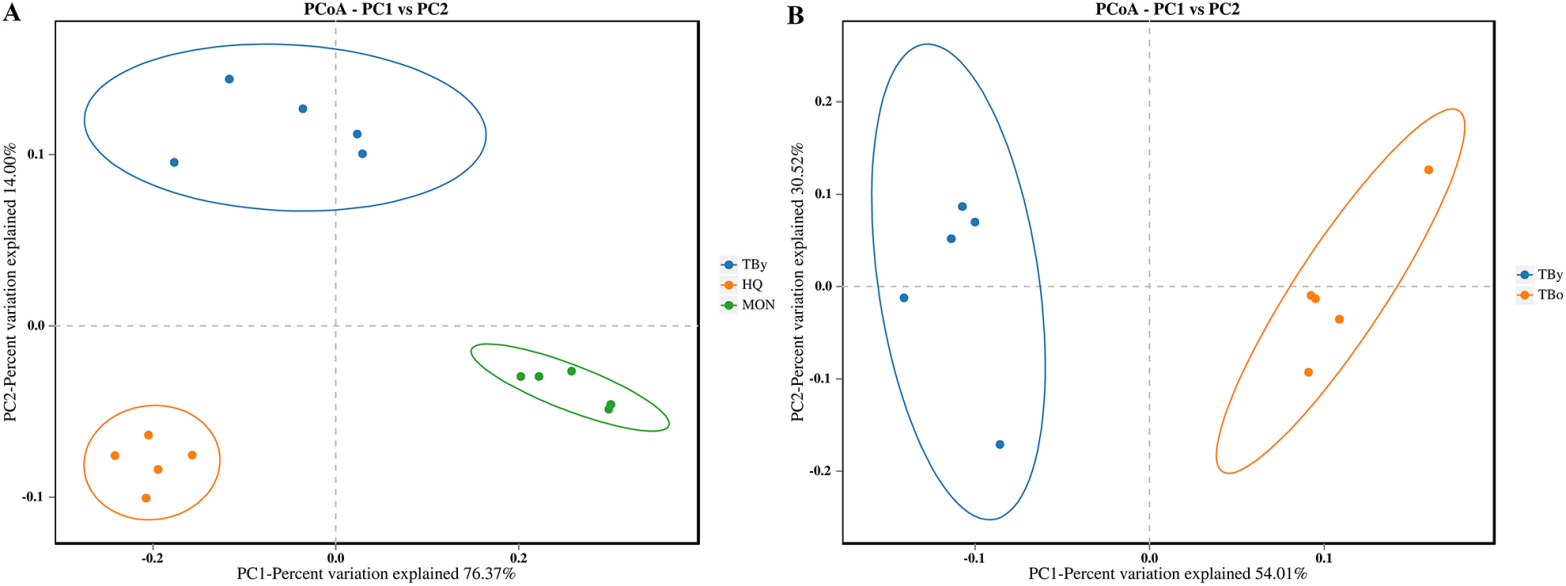
Differences in beta diversity between groups. **(A)** PCoA analysis among the breed groups. **(B)** PCoA analysis between the age groups. TBy, The younger Thoroughbred horses; TBo, The older Thoroughbred horses; HQ, Hequ horses; MON, Mongolian horses. Statistic algorithm, weighted unifrac algorithm.

The Good’s coverage of all clone libraries was 1.0, indicating that the sequencing results could accurately represent the actual microbiota composition in the samples. Among the three breeds, the ACE and Simpson indices were highest in the HQ group, followed by the TBy and MON groups (*P*<0.01) (**Figures 2A, B**). Conversely, neither the ACE index nor the Simpson index varied significantly between the two age groups of Thoroughbred horses (*P*>0.05) (**Figures 2C, D**). The PCoA plot was constructed based on the weighted uniFrac distance matrix, and the similarity between groups was verified by PERMANOVA (using weighted uniFrac). The results demonstrated that samples of the same breed or age clustered together, with significant differences between breed groups (*R* ^2^ = 0.852, *P*=0.001) and age groups (*R* ^2^ = 0.527, *P*=0.01) (**Figure 3**).

### The association between breed and gut microbiota of horses

**Figure 4:**
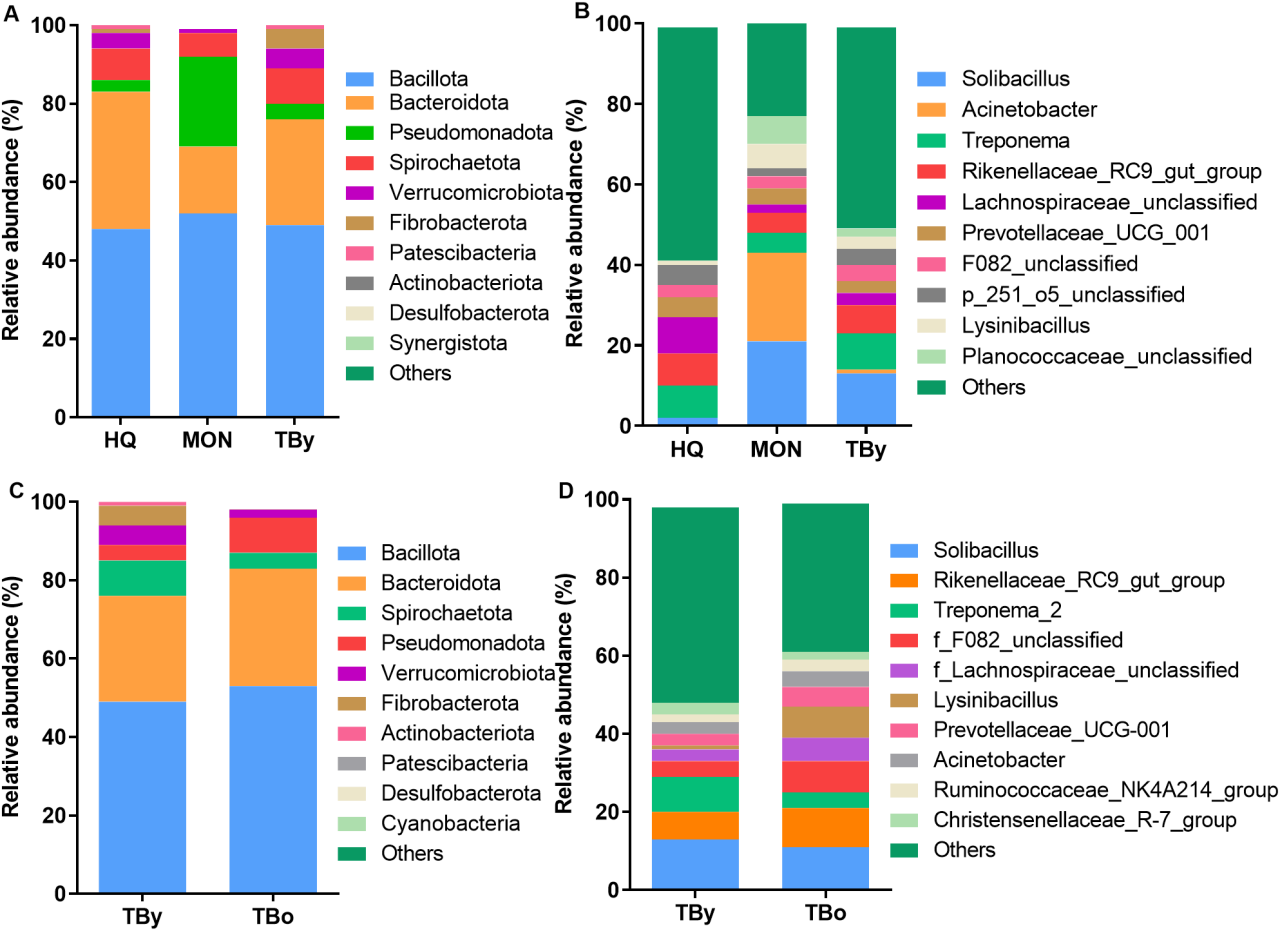
Analysis of the taxa differences between the groups. **(A)** The distribution histogram of the top ten phyla in the breed groups. **(B)** The distribution histogram of the top ten genera in the breed groups. **(C)** The distribution histogram of the top ten phyla in the age groups. **(D)** The distribution histogram of the top ten genera in the age groups. TBy, The younger Thoroughbred horses; TBo, The older Thoroughbred horses; HQ, Hequ horses; MON, Mongolian horses.

**Figure 5:**
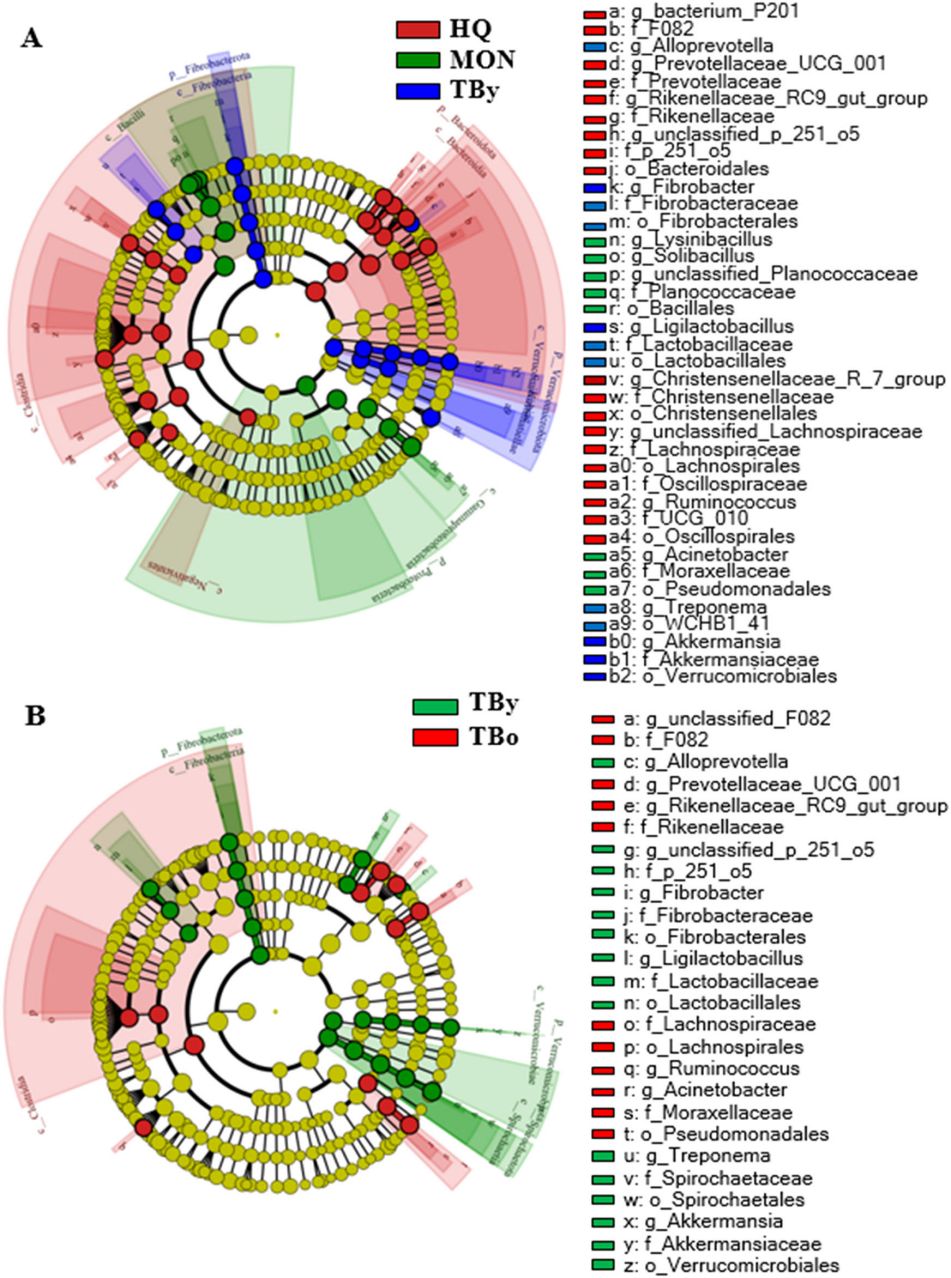
Cladogram based on LEfSe analysis. **(A)** LEfSe cladogram of the breed groups. **(B)** LEfSe cladogram of the age groups. TBy, The younger Thoroughbred horses; TBo, The older Thoroughbred horses; HQ, Hequ horses; MON, Mongolian horses.

In all samples, the predominant phylum was Bacillota, representing 47.57% for the HQ group, 52.31% for the MON group, and 48.57% for the TBy group, respectively (**Figure 4A**). Differential analysis between groups showed significant differences in 69.23% of the annotated phyla, including Bacteroidota, Verrucomicrobiota, Pseudomonadota, Fibrobacterota, Synergistota, Desulfobacterota, Actinobacteriota, and Deinococcota (**Supplementary Table S2**). At the genus level, the composition of the gut microbiota also varied significantly. The predominant genera of the HQ group were *Lachnospiraceae_unclassified* and *Rikenellaceae_RC9_gut_group*, the MON group were *Acinetobacter* and *Solibacillus*, and the TBy group were *Solibacillus* and *Treponema*, respectively (**Figure 4B**). Among the annotated 146 genera, 8.90% of genera in the HQ group were significantly different from the other two groups, 7.53% of genera in the MON group were significantly different from the other two groups, and 2.05% of genera in the TBy group were significantly different from the other two groups (**Supplementary Table S3**). Notably, the B/B ratios were 1.38, 3.15, 1.83, and 1.79 in the HQ, MON, TBy, and TBo groups, respectively. The LEfSe analysis showed that 51 taxa varied significantly among the three breeds (*P*<0.05). At the genus level, biomarkers enriched in the HQ group included *Lachnospiraceae_unclassified*, *bacterium_P201*, *f_p_251_o5_unclassified*, *Ruminococcus*, *Rikenellaceae_RC9_gut_group*, *Christensenellaceae_R_7_group*, and *Prevotellaceae_UCG_001*. The MON group contained four biomarkers, i.e. *Solibacillus*, *Acinetobacter*, *Lysinibacillus*, and *Planococcaceae_unclassified*. Biomarkers enriched in the TBy group were *Fibrobacter*, *Treponema*, *Ligilactobacillus*, *Alloprevotella*, and *Akkermansia* (**Figure 5A**).

### The association between age and gut microbiota of Thoroughbred horses

The predominant phyla in the TBy and TBo group were Bacillota (TBy: 48.58%; TBo: 53.24%) and Bacteroidota (TBy: 27.22%; TBo: 30.37%), with no significant difference in their relative abundance (**Figure 4C**). However, Spirochaetota, Fibrobacterota, Verrucomicrobiota, Patescibacteria, and Synergistota differed in abundance between the two groups (*P*<0.05) (**Supplementary Table S4**). At the genus level, *Solibacillus* was the predominant genus in the TBy and TBo groups with no significant difference (13.18% vs. 11.08%, *P*>0.05) (**Figure 4D**). A comparison of the relative abundances showed that 22.48% of the genera were significantly different between the TBy and TBo groups (**Supplementary Table S5**). LEfSe analysis found the biomarkers with the greatest effect on group differences. At the genus level, *Treponema*, *Fibrobacter*, *p_251_o5_unclassified*, *Ligilactobacillus, Alloprevotella*, and *Akkermansia* were biomarkers of the TBy group. The biomarkers of the TBo group were *f_F082_unclassified*, *Acinetobacter*, *Rikenellaceae_RC9_gut_group*, *Prevotellaceae_UCG_001*, and *Ruminococcus* (**Figure 5B**).

### Differences in KEGG metabolic pathways between groups

**Figure 6:**
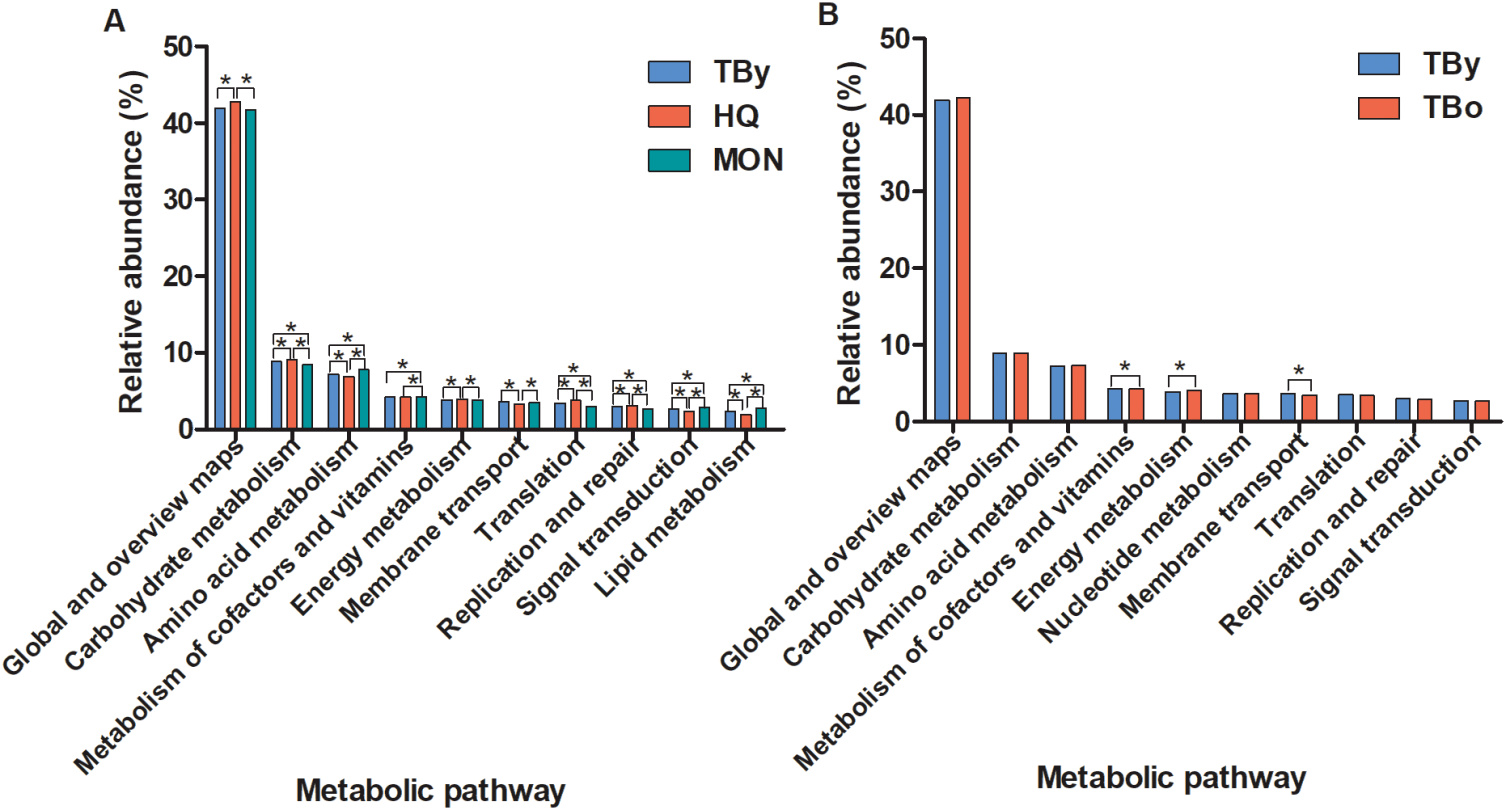
Differences of the KEGG metabolic pathways between groups. **(A)** The top ten pathways differences between the breed groups. **(B)** The top ten pathways differences between the age groups. TBy, The young Thoroughbred horses; TBo, The older Thoroughbred horses; HQ, Hequ horses; MON, Mongolian horses. Statistic analysis: One-way ANOVA with Tukey’s post test for the three breed groups; Student’s t-test for the two age groups. *P < 0.05.

PICRUSt2 software was used to perform predictive analysis based on the KEGG database to discover differential metabolic pathways in the gut microbiota between groups. The top 10 metabolic pathways that differed significantly between the breed groups were shown in **Figure 6A**. Among these metabolic pathways, carbohydrate metabolism, nucleotide metabolism, biosynthesis of other secondary metabolites, as well as global and overview maps were significantly enriched in the HQ group. Lipid metabolism, amino acid metabolism, metabolism of terpenoids and polyketides, xenobiotics biodegradation and metabolism, as well as metabolism of other amino acids were significantly enriched in the MON group. No metabolic pathways were found significantly higher in the TBy group than in the HQ and MON groups. In contrast, only two metabolic pathways (i.e., metabolism of cofactors and vitamins, and energy metabolism) were significantly different between the two age groups of Thoroughbred horses, and both differential metabolic pathways were significantly enriched in the TBo group (**Figure 6B**).

## Discussion

The ACE and Simpson indices are indicators reflecting the richness and evenness of the microbiota in the sequenced samples. A comparison of the ACE and Simpson indices across breed groups showed that the Hequ horses had the richest and most diverse gut microbiota, followed by the Thoroughbred and Mongolian horses, with significant differences across horse breeds. Bacillota was the predominant phylum in all groups, which is consistent with previous studies on gut microbiota of horses (Adams et al., 2022; Wang et al., 2024). Most bacteria within the Bacillota possess the ability to ferment carbohydrates to generate butyrate. This process is crucial as butyrate provides energy and stimulates the development of host intestinal epithelial cells (Yang et al., 2019). In the animal gut, Bacillota and Bacteroidota coexist symbiotically. Both are essential for fermenting polysaccharides, and a stable B/B ratio is of great significance for host health (Kmezik et al., 2021). The B/B ratio regulates the host gut microbiota composition and carbohydrate metabolism (Rehman et al., 2022). For example, an elevated B/B ratio could improve the absorption of feed nutrients and cause obesity in horses (Biddle et al., 2018; Morrison et al., 2018). Jiang et al. (2021) found that the gut microbiota of Musk deer has a higher B/B ratio in the cold season, which improved the cold tolerance of Musk deer by promoting the decomposition of cellulose and hemicellulose, the metabolism of carbohydrates and the digestion and absorption of nutrients (Jiang et al., 2021). In the present study, the B/B ratio in the gut microbiota of the Mongolian horses was significantly higher than that of the other two breeds, which may be related to its roughage and cold resistance.

Biomarkers enriched in the gut of the Hequ horses were composed primarily of commensal bacteria from Bacillota and Bacteroidota. At the genus level, biomarkers from Bacillota included *Lachnospiraceae_unclassified*, *Ruminococcus*, and *Christensenellaceae_R_7_group*, all of which have the ability to help the host obtain energy from dietary fibers. The genome of Lachnospiraceae contains varied genes encoding carbohydrate-degrading enzymes and has considerable cellulolytic ability to participate in host metabolism through fermentation products such as butyrate and other SCFAs (Petry et al., 2021; Ayoub et al., 2022; Cho et al., 2024). *Ruminococcus* also has strong fiber degradation ability, and positively associate with feed utilization and SCFAs metabolism of host (Mach et al., 2021). *Christensenellaceae_R_7_group* is not only involved in amino acid and lipid metabolism, but its relative abundance is also positively correlated with acetate and butyrate in the cecum of host (Waters and Ley, 2019; Dong et al., 2022). The genus-level biomarkers belonging to Bacteroidota included *bacterium_P201*, *Prevotellaceae_UCG_001*, *Rikenellaceae_RC9_gut_group*, and *p_251_o5_unclassified*. These biomarkers are capable of fermenting polysaccharides to produce SCFAs. The family F082, to which *bacterium_P201* belongs, was verified to be associated with propionic acid, propionate, and NH_3_-N metabolism in the sheep rumen (Han et al., 2022; Jize et al., 2022). *Prevotellaceae_UCG_001* has enzymes that degrade cellulose and xylose, which was confirmed to be positively associated with AMPK signaling, and negatively associated with markers of the glycolipid metabolism disorder (Song et al., 2019). *Rikenellaceae_RC9_gut_group* is able to ferment carbohydrates to produce SCFAs such as acetate, propionate, butyrate, and succinate, and its abundance was positively correlated with the proportion of roughage added in the feed (Ahmad et al., 2022). A comparison of the KEGG metabolic pathways across breeds revealed that the gut microbiota of the Hequ horses is rich in genes involved in carbohydrate metabolism and glycan biosynthesis and metabolism, further validating the involvement of these biomarkers in horse metabolism. Enriched bacterial communities involved in the utilization of dietary fibers suggest that the Hequ horses are capable of efficiently digesting the roughage in its diet. Based on this consideration, it is conjectured in the current study that the proportion of roughage in the diet of the Hequ horses may be suitably increased.

Planococcaceae (*Solibacillus*), Bacillaceae (*Lysinibacillus*), and Moraxellaceae (*Acinetobacter*) were enriched in the MON group. *Solibacillus* sp. and *Lysinibacillus* sp. possess a large number of bile salt metabolism genes. These genes can degrade conjugated bile salts to generate secondary bile acids, and then promote lipid digestion and absorption (Yan et al., 2021). In addition, members of the genus *Acinetobacter* are also involved in cofactors and vitamins metabolism and amino acid metabolism in the gut of host (Zhao et al., 2022). Thus, it is believed that *Solibacillus* and *Lysinibacillus* are the main genera responsible for lipid metabolism and amino acid metabolism enrichment in the gut of the Mongolian horses. The highest B/B ratio and the enrichment of bacterial communities involved in the metabolism of bile acids, lipids and amino acids in the gut of the Mongolian horses resulted in significantly higher lipid metabolism and amino acid metabolism than in the other two breeds. It is well known that the genus *Acinetobacter* contains many opportunistic pathogens, such as *A. baumannii*, *A. nosocomialis*, and *A. calcoaceticus*, which can cause a variety of diseases (Kim et al., 2016; van der Kolk et al., 2019). However, certain species of *Acinetobacter* (PDSCDXS_12C and L9B) have also demonstrated high-efficiency cellulose-degradation capabilities in the insect gut (Pourramezan et al., 2012; Zhang et al., 2020; Akter et al., 2021). Meanwhile, at least 20 CAZy families participating in hemicellulose degradation have been identified in the genome of *Acinetobacter* (Jin et al., 2021). Given this, the function of the *Acinetobacter* significantly enriched in MON samples warrants further investigation. Notably, the gut microbiota of Mongolian horses has the highest B/B ratio, and contains the bacterial community associated with the bile salt metabolism, which is in line with the characteristics of rapid fattening of Mongolian horses (Ai and Wang, 2019). As a result, the supply ratio of concentrate or energy feed needs to be controlled to prevent obesity in Mongolian horses.

The genus-level biomarkers enriched in the gut of Thoroughbred horses included *Fibrobacter*, *Treponema*, *Ligilactobacillus*, *Alloprevotella*, and *Akkermansia*. *Fibrobacter* is capable of fermenting cellulose and cellobiose to produce acetic and succinic acid (Kobayashi et al., 2020). *Treponema* participates in the degradation of pectin in roughage and promotes protein synthesis, which is beneficial to the improvement of animal performance (Liu et al., 2014; Lv et al., 2023). *Alloprevotella* is also a SCFAs producing genus (Qu et al., 2017). Bacteria of the genus *Ligilactobacillus* are able to promote the recovery of intestinal microbiota structure and intestinal barrier function in the host, and have potential anti-inflammatory effects (Fei et al., 2022; Li et al., 2022b; Xie et al., 2024). *Akkermansia* can not only ferment carbohydrates to produce SCFAs, but also protect the gut from pathogens through competitive exclusion (Lindenberg et al., 2021; Ma et al., 2023). The enrichment of these biomarkers suggests that Thoroughbred horses have a good carbohydrate metabolic capacity and better intestinal barrier function. However, based on the relatively low level of energy metabolism in Thoroughbred horses, energy feed supplies should be guaranteed during feeding. Notably, the disparities in alpha diversity, B/B ratio, dominant bacterial communities, and enriched bacterial functions between Mongolian and Thoroughbred horses align with the previous study (Wen et al., 2022), underscoring a close association between horse breed and gut microbiota composition. However, nuanced distinctions exist between the two studies. Specifically, this study revealed significantly higher relative abundances of Pseudomonadota and Deinococcota in Mongolian horses compared to Thoroughbred horses. While Actinobacteriota did not differ significantly between breeds, Verrucomicrobiota exhibited a notably higher relative abundance in Thoroughbred horses than in Mongolian horses. In functional prediction analyses, the proportion of gut microbial functions related to lipid metabolism was significantly greater in Mongolian horses than in Thoroughbred horses. These inter-study discrepancies may be attributed to variations in rearing environments, exercise regimes, and feed sources.

Association analysis of age and gut microbiota of Thoroughbred horses showed no significant differences in the alpha diversity of gut microbiota between younger and older groups. The study revealed that the diversity of gut microbiota in Thoroughbred foals under one year of age gradually increases with age and eventually stabilizes (Leng et al., 2024). However, consistent with previous studies, the composition and function of gut microbiota differed across different age stages (De La Torre et al., 2019; Baraille et al., 2024). The two age groups were composed of different glycolytic bacteria. At the genus level, *Fibrobacter*, *Ligilactobacillus*, *Alloprevotella*, and *Akkermansia* were enriched in the gut of younger Thoroughbred horses. *F082_unclassified*, *Rikenellaceae_RC9_gut_group*, *Ruminococcus*, and *Prevotellaceae_UCG_001* were enriched in the gut of older Thoroughbred horses. However, microbial genes involved in carbohydrate metabolism and glycan biosynthesis and metabolism did not differ significantly between the two age groups. Enriched metabolism of cofactors and vitamins, as well as increased energy metabolism in the gut of older Thoroughbred horses, suggest a more vigorous capacity to utilize these nutrients. Based on the differences in gut microbiota between the two age groups, the current study speculates that the proportion of vitamins and energy feed in the diet should be adjusted based on the age of the Thoroughbred horses. Limitations of this study include the small sample size, the single sampling during the trial period, and the limitations of amplicon-based functional prediction in identifying strain-specific and rare environment-specific functions. In the following studies, more meaningful data will be acquired by expanding the sample size, increasing the sampling frequency, and employing shotgun metagenomics in conjunction with metabolomics.

## Conclusion

In summary, this study comprehensively analyzed the gut microbiota of three horse breeds and two age groups of Thoroughbred horses to explore the associations between horse breed or age and gut microbiota. The differential analysis of gut microbiota among breeds showed significant differences in alpha diversity, beta diversity, composition, and the involved metabolic pathways. When comparing the gut microbiota differences between the two age groups of Thoroughbred horses, no significant differences were detected in alpha diversity, the B/B ratio, and 83.33% of the metabolic pathways. The main differences between the two groups were manifested in two aspects: they consisted of different glycolytic bacteria, and there were significant differences in cofactor and vitamin metabolism, as well as energy metabolism. The differential biomarkers and metabolic pathways identified through the association analysis of breed and gut microbiota, or age and gut microbiota, can be utilized to improve equine gut microbiota databases and facilitate the development of scientific feeding strategies. For instance, since Hequ horses have a greater fiber-degradation capacity, the proportion of coarse fiber in their diet can be increased. The enrichment of bacterial communities related to lipid digestion and absorption in the gut of Mongolian horses, along with their rapid-fattening characteristics, necessitates our attention to controlling the proportion of concentrated or energy-rich feed in the diet to prevent obesity. Conversely, as the proportion of bacterial communities involved in energy metabolism is relatively low in the gut of Thoroughbred horses, an adequate supply of concentrates or energy-rich feeds should be ensured in their diet. Given that the gut microbiota involved in cofactor and vitamin metabolism, as well as energy metabolism, varies significantly between younger and older Thoroughbred horses, the supply of cofactors, vitamins, and energy substances needs to be adjusted according to the Thoroughbred horse’s age.

## Data Availability

The datasets presented in this study can be found in online repositories. The names of the repository/repositories and accession number(s) can be found below: https://www.ncbi.nlm.nih.gov/, PRJNA887344.
