# Supplementary material for: Exploring the distinctive characteristics of gut microbiota across different horse breeds and ages using metataxonomics
Source: Front Cell Infect Microbiol. 2025 Jul 7;15:1590839. doi: 10.3389/fcimb.2025.1590839 (PMC12277257; doi:10.3389/fcimb.2025.1590839)
Supplement: Supplementary file 3 [file DataSheet3.docx]

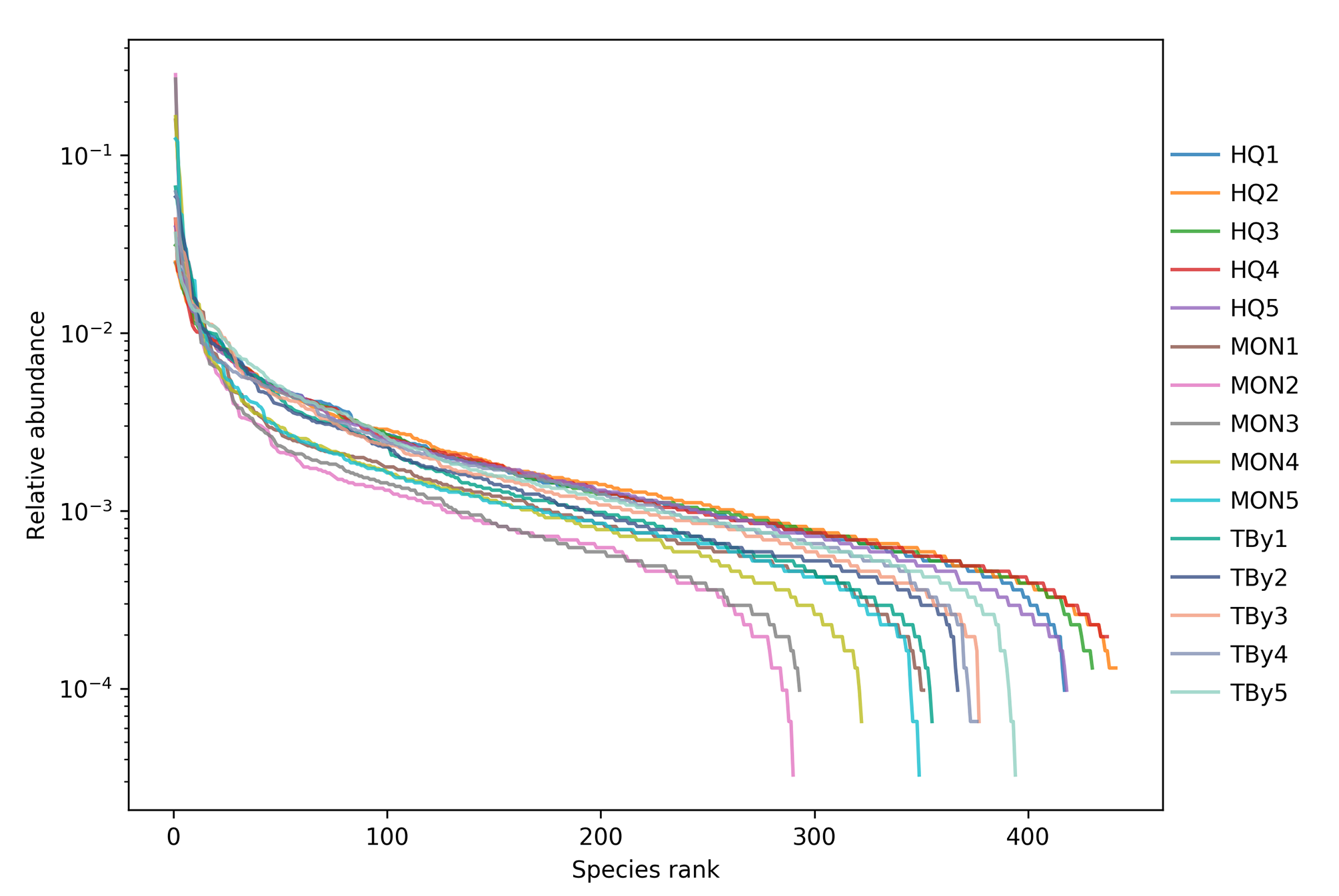


Supplementary Figure S3 Rank abundance curves for the three breed groups. TBy: The younger Thoroughbred horses. HQ: Hequ horses. MON: Mongolian horses.
