# Supplementary material for: Exploring the distinctive characteristics of gut microbiota across different horse breeds and ages using metataxonomics
Source: Front Cell Infect Microbiol. 2025 Jul 7;15:1590839. doi: 10.3389/fcimb.2025.1590839 (PMC12277257; doi:10.3389/fcimb.2025.1590839)
Supplement: Supplementary file 5 [file Table1.docx]

Supplementary Table S1 Raw data processing results

| Sample ID | Raw Reads | Clean Reads | Denoised Reads | Merged Reads | Non-chimeric Reads |
| --- | --- | --- | --- | --- | --- |
| HQ1 | 80,016 | 51,150 | 47,881 | 40,759 | 34,781 |
| HQ2 | 80,185 | 51,324 | 48,192 | 41,603 | 36,192 |
| HQ3 | 80,004 | 51,228 | 48,231 | 40,886 | 34,108 |
| HQ4 | 80,239 | 50,665 | 48,056 | 41,555 | 36,122 |
| HQ5 | 80,159 | 50,717 | 47,943 | 41,364 | 35,466 |
| MON1 | 79,689 | 51,030 | 48,760 | 44,573 | 38,639 |
| MON2 | 80,134 | 51,572 | 49,877 | 46,242 | 38,888 |
| MON3 | 79,705 | 51,240 | 49,471 | 45,886 | 38,075 |
| MON4 | 80,230 | 51,692 | 49,429 | 45,006 | 36,553 |
| MON5 | 80,275 | 51,130 | 48,988 | 44,558 | 36,964 |
| TBy1 | 80,123 | 51,046 | 48,629 | 42,490 | 33,133 |
| TBy2 | 79,816 | 50,886 | 48,362 | 42,149 | 33,377 |
| TBy3 | 80,149 | 51,553 | 48,936 | 42,022 | 34,008 |
| TBy4 | 80,328 | 51,083 | 48,316 | 41,155 | 34,862 |
| TBy5 | 79,748 | 50,653 | 47,803 | 41,415 | 35,477 |
| TBo1 | 80,058 | 50,971 | 48,636 | 42,000 | 34,041 |
| TBo2 | 80,115 | 51,395 | 49,112 | 42,778 | 35,529 |
| TBo3 | 80,351 | 51,440 | 49,081 | 42,480 | 34,912 |
| TBo4 | 79,757 | 51,117 | 48,820 | 42,328 | 35,618 |
| TBo5 | 80,160 | 51,229 | 49,644 | 44,800 | 39,001 |
