# Supplementary material for: Exploring the distinctive characteristics of gut microbiota across different horse breeds and ages using metataxonomics
Source: Front Cell Infect Microbiol. 2025 Jul 7;15:1590839. doi: 10.3389/fcimb.2025.1590839 (PMC12277257; doi:10.3389/fcimb.2025.1590839)
Supplement: Supplementary file 6 [file Table2.docx]

Supplementary Table S2 Differences in the relative abundance of phyla between breeds (Kruskal Wallis rank-sum test, and *P*-values were corrected using the Benjamini-Hochberg method).

| Phylum | HQ  (%) | MON  (%) | TBy  (%) | HQ vs  MON (*P*) | HQ vs  TBy (*P*) | MON vs TBy(*P*) |
| --- | --- | --- | --- | --- | --- | --- |
| Bacillota | 47.57 | 52.31 | 48.57 |  |  |  |
| Bacteroidota | 34.69 | 17.00 | 27.26 | 0.002 | 0.266 | 0.185 |
| Spirochaetota | 8.33 | 5.77 | 9.27 |  |  |  |
| Verrucomicrobiota | 3.98 | 0.81 | 4.86 | 0.018 | 0.939 | 0.047 |
| Pseudomonadota | 2.87 | 22.83 | 3.71 | 0.018 | 0.939 | 0.047 |
| Fibrobacterota | 0.71 | 0.26 | 4.97 | 0.209 | 0.209 | 0.002 |
| Patescibacteria | 0.65 | 0.45 | 0.51 |  |  |  |
| Synergistota | 0.43 | 0.15 | 0.00 | 0.198 | 0.002 | 0.198 |
| Desulfobacterota | 0.26 | 0.09 | 0.23 | 0.013 | 0.851 | 0.060 |
| Campylobacterota | 0.25 | 0.01 | 0.07 | 0.002 | 0.209 | 0.209 |
| Actinobacteriota | 0.23 | 0.16 | 0.50 | 0.914 | 0.066 | 0.022 |
| Deinococcota | 0.00 | 0.14 | 0.00 | 0.045 | 1.000 | 0.045 |
| unclassified_Bacteria | 0.00 | 0.00 | 0.00 |  |  |  |
