# Supplementary material for: Exploring the distinctive characteristics of gut microbiota across different horse breeds and ages using metataxonomics
Source: Front Cell Infect Microbiol. 2025 Jul 7;15:1590839. doi: 10.3389/fcimb.2025.1590839 (PMC12277257; doi:10.3389/fcimb.2025.1590839)
Supplement: Supplementary file 7 [file Table3.docx]

Supplementary Table S3 Differences in the relative abundance of genera between breeds (Kruskal Wallis rank-sum test, and *P*-values were corrected using the Benjamini-Hochberg method).

| Genus | HQ  (%) | | MON  (%) | TBy  (%) | HQ vs  MON (*P*) | HQ vs  TBy (*P*) | MON vs TBy(*P*) |
| --- | --- | --- | --- | --- | --- | --- | --- |
| *Solibacillus* | 1.55 | | 21.07 | 13.19 | 0.004 | 0.141 | 0.406 |
| *Treponema* | 7.73 | | 5.38 | 8.91 |  |  |  |
| *Rikenellaceae_RC9_gut_group* | 8.09 | 5.05 | | 6.90 | 0.004 | 0.406 | 0.141 |
| *Fibrobacter* | 0.71 | | 0.26 | 4.97 | 0.209 | 0.209 | 0.002 |
| *unclassified_F082* | 3.39 | | 3.07 | 4.38 |  |  |  |
| *unclassified_p_251_o5* | 5.34 | | 1.55 | 3.57 | 0.002 | 0.210 | 0.210 |
| *Lysinibacillus* | 0.68 | | 5.76 | 3.27 | 0.005 | 0.367 | 0.184 |
| *Prevotellaceae_UCG_001* | 5.38 | | 3.56 | 3.19 | 0.047 | 0.018 | 0.939 |
| *unclassified_Lachnospiraceae* | 8.82 | | 2.44 | 3.13 | 0.012 | 0.066 | 0.817 |
| *uncultured_rumen_bacterium* | 3.12 | | 1.28 | 3.09 | 0.018 | 0.939 | 0.047 |
| *Ligilactobacillus* | 0.67 | | 0.07 | 2.77 | 0.209 | 0.209 | 0.002 |
| *Alloprevotella* | 0.14 | | 0.05 | 2.53 | 0.444 | 0.131 | 0.004 |
| *Lachnospiraceae_XPB1014_group* | 2.14 | | 1.66 | 2.41 |  |  |  |
| *unclassified_Planococcaceae* | 0.30 | | 7.04 | 2.35 | 0.002 | 0.210 | 0.210 |
| *Akkermansia* | 1.66 | | 0.23 | 2.22 | 0.027 | 0.885 | 0.090 |
| *NK4A214_group* | 3.14 | | 1.24 | 2.07 | 0.002 | 0.210 | 0.210 |
| *Escherichia_Shigella* | 2.26 | | 0.29 | 2.04 |  |  |  |
| *Phascolarctobacterium* | 1.96 | | 0.20 | 1.86 | 0.022 | 0.978 | 0.039 |
| *unclassified_Muribaculaceae* | 0.67 | | 0.47 | 1.66 | 0.486 | 0.122 | 0.005 |
| *Prevotellaceae_UCG_004* | 1.41 | | 1.29 | 1.63 |  |  |  |
| *UCG_005* | 1.67 | | 0.25 | 1.62 | 0.015 | 0.885 | 0.056 |
| *unclassified_[Eubacterium]_coprostanoligenes_group* | 2.34 | | 1.56 | 1.60 | 0.090 | 0.077 | 0.997 |
| *Acinetobacter* | 0.42 | | 21.80 | 1.35 | 0.004 | 0.406 | 0.141 |
| *UCG_002* | 1.48 | | 0.44 | 1.31 | 0.015 | 0.885 | 0.056 |
| *Lachnospiraceae_AC2044_group* | 0.59 | | 0.91 | 1.29 | 0.184 | 0.005 | 0.367 |
| *Christensenellaceae_R_7_group* | 3.54 | | 1.24 | 1.27 | 0.033 | 0.027 | 0.998 |
| *unclassified_Ruminococcaceae* | 0.81 | | 0.47 | 1.25 | 0.105 | 0.570 | 0.006 |
| *Prevotella* | 1.09 | | 0.11 | 1.01 | 0.012 | 0.817 | 0.066 |
| *unclassified_UCG_010* | 1.05 | | 0.25 | 0.82 | 0.010 | 0.739 | 0.077 |
| *Saccharofermentans* | 1.39 | | 0.43 | 0.81 | 0.002 | 0.209 | 0.209 |
| *unclassified_Bacteroidales* | 0.76 | | 0.30 | 0.70 | 0.018 | 0.939 | 0.047 |
| *Anaerovorax* | 1.07 | | 0.23 | 0.69 | 0.006 | 0.570 | 0.105 |
| *unclassified_WCHB1_41* | 0.84 | | 0.22 | 0.69 | 0.022 | 0.914 | 0.066 |
| *Candidatus_Saccharimonas* | 0.64 | | 0.11 | 0.51 | 0.011 | 0.778 | 0.071 |
| *unclassified_Clostridia_UCG_014* | 0.81 | | 0.26 | 0.44 | 0.010 | 0.122 | 0.613 |
| *Lachnospiraceae_UCG_009* | 1.70 | | 1.13 | 0.42 | 0.210 | 0.002 | 0.210 |
| *hoa5_07d05_gut_group* | 0.23 | | 0.21 | 0.38 | 0.885 | 0.141 | 0.047 |
| *Oribacterium* | 0.59 | | 0.30 | 0.37 | 0.027 | 0.033 | 0.998 |
| *Defluviitaleaceae_UCG_011* | 0.58 | | 0.11 | 0.37 | 0.002 | 0.210 | 0.210 |
| *Family_XIII_AD3011_group* | 0.30 | | 0.11 | 0.36 | 0.077 | 0.739 | 0.010 |
| *Prevotellaceae_UCG_003* | 0.70 | | 0.05 | 0.33 | 0.002 | 0.210 | 0.210 |
| *Sphaerochaeta* | 0.24 | | 0.15 | 0.31 |  |  |  |
| *Lactobacillus* | 0.35 | | 0.58 | 0.30 |  |  |  |
| *unclassified_Christensenellaceae* | 0.26 | | 0.38 | 0.29 |  |  |  |
| *unclassified_Coriobacteriales_Incertae_Sedis* | 0.01 | | 0.05 | 0.28 | 0.402 | 0.004 | 0.138 |
| *unclassified_Bacteroidales_UCG_001* | 0.25 | | 0.03 | 0.27 | 0.024 | 0.990 | 0.035 |
| *Quinella* | 0.23 | | 0.12 | 0.26 |  |  |  |
| *unclassified_Erysipelotrichaceae* | 0.22 | | 0.07 | 0.26 | 0.077 | 0.852 | 0.018 |
| *Desulfovibrio* | 0.20 | | 0.09 | 0.23 | 0.046 | 0.939 | 0.018 |
| *unclassified_Oscillospirales* | 0.09 | | 0.01 | 0.22 | 0.209 | 0.209 | 0.002 |
| *Faecalibacterium* | 0.06 | | 0.01 | 0.21 | 0.563 | 0.101 | 0.006 |
| *unclassified_Eggerthellaceae* | 0.05 | | 0.01 | 0.19 | 0.637 | 0.077 | 0.006 |
| *Ruminiclostridium* | 0.09 | | 0.09 | 0.18 |  |  |  |
| *Candidatus_Soleaferrea* | 0.20 | | 0.11 | 0.18 |  |  |  |
| *unclassified_Bacteroidales_BS11_gut_group* | 1.05 | | 0.13 | 0.17 | 0.012 | 0.066 | 0.817 |
| *Bacteroides* | 0.15 | | 0.02 | 0.17 | 0.044 | 0.990 | 0.030 |
| *unclassified_Oscillospiraceae* | 0.26 | | 0.05 | 0.15 | 0.004 | 0.405 | 0.140 |
| *Agathobacter* | 0.00 | | 0.00 | 0.15 | 1.000 | 0.007 | 0.007 |
| *Kurthia* | 0.00 | | 0.00 | 0.14 | 1.000 | 0.075 | 0.075 |
| *unclassified_Rikenellaceae* | 0.68 | | 0.00 | 0.14 | 0.002 | 0.197 | 0.197 |
| *Incertae_Sedis* | 0.12 | | 0.02 | 0.13 | 0.065 | 0.816 | 0.012 |
| *Monoglobus* | 0.59 | | 0.05 | 0.12 | 0.006 | 0.105 | 0.569 |
| *unclassified_Prevotellaceae* | 0.69 | | 0.12 | 0.12 | 0.027 | 0.033 | 0.998 |
| *Lachnospiraceae_UCG_008* | 0.12 | | 0.00 | 0.11 | 0.080 | 1.000 | 0.080 |
| *FD2005* | 0.14 | | 0.05 | 0.10 |  |  |  |
| *Ruminococcus* | 2.38 | | 0.97 | 0.10 | 0.210 | 0.002 | 0.210 |
| *unclassified_Bacteroidia* | 0.06 | | 0.24 | 0.09 |  |  |  |
| *Catenisphaera* | 0.02 | | 0.00 | 0.09 | 0.448 | 0.153 | 0.006 |
| *unclassified_Clostridia* | 0.40 | | 0.81 | 0.08 | 0.209 | 0.209 | 0.002 |
| *UCG_007* | 0.01 | | 0.00 | 0.07 |  |  |  |
| *unclassified_Rhodospirillales* | 0.04 | | 0.00 | 0.07 | 0.386 | 0.449 | 0.030 |
| *unclassified_Xanthobacteraceae* | 0.04 | | 0.01 | 0.07 |  |  |  |
| *UCG_009* | 0.01 | | 0.00 | 0.07 | 0.856 | 0.047 | 0.010 |
| *unclassified_Peptococcaceae* | 0.06 | | 0.03 | 0.06 |  |  |  |
| *Caryophanon* | 0.00 | | 0.00 | 0.05 |  |  |  |
| *Actinobacillus* | 0.00 | | 0.00 | 0.05 |  |  |  |
| *unclassified_Spirochaetaceae* | 0.36 | | 0.24 | 0.05 | 0.332 | 0.003 | 0.162 |
| *unclassified_Oxalobacteraceae* | 0.00 | | 0.00 | 0.04 | 1.000 | 0.024 | 0.024 |
| *Campylobacter* | 0.04 | | 0.01 | 0.04 |  |  |  |
| *[Anaerorhabdus]_furcosa_group* | 0.00 | | 0.00 | 0.04 | 0.898 | 0.082 | 0.026 |
| *Streptococcus* | 1.09 | | 0.19 | 0.04 | 0.208 | 0.002 | 0.208 |
| *Erysipelatoclostridium* | 0.00 | | 0.01 | 0.04 | 0.680 | 0.025 | 0.186 |
| *unclassified_Gastranaerophilales* | 0.25 | | 0.01 | 0.04 | 0.005 | 0.120 | 0.483 |
| *[Eubacterium]_ruminantium_group* | 0.00 | | 0.00 | 0.04 |  |  |  |
| *Bifidobacterium* | 0.00 | | 0.00 | 0.03 |  |  |  |
| *Shuttleworthia* | 0.02 | | 0.00 | 0.03 |  |  |  |
| *Bradyrhizobium* | 0.00 | | 0.00 | 0.02 |  |  |  |
| *[Eubacterium]_siraeum_group* | 0.02 | | 0.00 | 0.02 | 0.164 | 0.749 | 0.029 |
| *Oscillibacter* | 0.00 | | 0.00 | 0.02 |  |  |  |
| *UCG_004* | 0.06 | | 0.01 | 0.02 | 0.006 | 0.144 | 0.477 |
| *unclassified_Syntrophomonadaceae* | 0.01 | | 0.00 | 0.02 |  |  |  |
| *unclassified_Enterobacteriaceae* | 0.02 | | 0.07 | 0.02 | 0.045 | 0.990 | 0.031 |
| *Burkholderia_Caballeronia_Paraburkholderia* | 0.03 | | 0.00 | 0.02 |  |  |  |
| *Anaerofustis* | 0.02 | | 0.00 | 0.02 |  |  |  |
| *Mogibacterium* | 0.13 | | 0.20 | 0.02 | 0.815 | 0.064 | 0.012 |
| *Lachnospiraceae_UCG_006* | 0.09 | | 0.05 | 0.01 | 0.197 | 0.013 | 0.514 |
| *Clostridium_sensu_stricto_3* | 0.00 | | 0.00 | 0.01 |  |  |  |
| *unclassified_Bacteroidales_RF16_group* | 0.38 | | 0.08 | 0.01 | 0.203 | 0.002 | 0.203 |
| *Limosilactobacillus* | 0.12 | | 0.01 | 0.01 | 0.027 | 0.015 | 0.975 |
| *[Eubacterium]_hallii_group* | 0.06 | | 0.08 | 0.01 |  |  |  |
| *Butyrivibrio* | 0.00 | | 0.01 | 0.01 |  |  |  |
| *unclassified_Dysgonomonadaceae* | 0.00 | | 0.00 | 0.01 |  |  |  |
| *unclassified_Anaerovoracaceae* | 0.04 | | 0.01 | 0.01 |  |  |  |
| *Bacillus* | 0.00 | | 0.05 | 0.01 | 0.010 | 0.855 | 0.047 |
| *Schwartzia* | 0.19 | | 0.01 | 0.00 | 0.018 | 0.014 | 0.997 |
| *unclassified_Succinivibrionaceae* | 0.02 | | 0.00 | 0.00 |  |  |  |
| *Corynebacterium* | 0.00 | | 0.10 | 0.00 |  |  |  |
| *Pseudomonas* | 0.00 | | 0.10 | 0.00 |  |  |  |
| *Rubellimicrobium* | 0.00 | | 0.07 | 0.00 |  |  |  |
| *unclassified_Absconditabacteriales__SR1* | 0.00 | | 0.19 | 0.00 | 0.007 | 1.000 | 0.007 |
| *Oligella* | 0.00 | | 0.16 | 0.00 | 0.075 | 1.000 | 0.075 |
| *unclassified_Synergistaceae* | 0.43 | | 0.15 | 0.00 | 0.198 | 0.002 | 0.198 |
| *Truepera* | 0.00 | | 0.14 | 0.00 | 0.075 | 1.000 | 0.075 |
| *Moheibacter* | 0.00 | | 0.14 | 0.00 | 0.075 | 1.000 | 0.075 |
| *Pseudobutyrivibrio* | 0.02 | | 0.13 | 0.00 | 0.083 | 0.898 | 0.027 |
| *Membranicola* | 0.00 | | 0.11 | 0.00 | 0.025 | 1.000 | 0.025 |
| *unclassified_Flavobacteriaceae* | 0.00 | | 0.10 | 0.00 | 0.075 | 1.000 | 0.075 |
| *Galbibacter* | 0.00 | | 0.10 | 0.00 |  |  |  |
| *unclassified_Selenomonadaceae* | 0.26 | | 0.09 | 0.00 | 0.198 | 0.002 | 0.198 |
| *unclassified_Xanthomonadaceae* | 0.00 | | 0.09 | 0.00 | 0.075 | 1.000 | 0.075 |
| *Anaeroplasma* | 0.12 | | 0.07 | 0.00 | 0.392 | 0.003 | 0.131 |
| *unclassified_RF39* | 0.04 | | 0.06 | 0.00 |  |  |  |
| *Aequorivita* | 0.00 | | 0.06 | 0.00 |  |  |  |
| *Castellaniella* | 0.00 | | 0.05 | 0.00 |  |  |  |
| *Pedobacter* | 0.00 | | 0.05 | 0.00 | 0.075 | 1.000 | 0.075 |
| *Halomonas* | 0.00 | | 0.05 | 0.00 |  |  |  |
| *unclassified_Fodinicurvataceae* | 0.00 | | 0.05 | 0.00 | 0.075 | 1.000 | 0.075 |
| *unclassified_Hungateiclostridiaceae* | 0.01 | | 0.04 | 0.00 | 0.171 | 0.562 | 0.013 |
| *Aminobacter* | 0.00 | | 0.03 | 0.00 |  |  |  |
| *unclassified_Balneolaceae* | 0.00 | | 0.03 | 0.00 |  |  |  |
| *Altererythrobacter* | 0.00 | | 0.03 | 0.00 |  |  |  |
| *Brevundimonas* | 0.00 | | 0.02 | 0.00 | 0.025 | 1.000 | 0.025 |
| *bacterium_P201* | 4.05 | | 0.01 | 0.00 | 0.020 | 0.006 | 0.924 |
| *Peptococcus* | 0.00 | | 0.01 | 0.00 |  |  |  |
| *unclassified_Bacteria* | 0.00 | | 0.00 | 0.00 |  |  |  |
| *Weissella* | 0.27 | | 0.00 | 0.00 | 0.007 | 0.007 | 1.000 |
| *Rummeliibacillus* | 0.19 | | 0.00 | 0.00 | 0.007 | 0.007 | 1.000 |
| *unclassified_Paludibacteraceae* | 0.15 | | 0.00 | 0.00 | 0.007 | 0.007 | 1.000 |
| *Phoenicibacter* | 0.14 | | 0.00 | 0.00 | 0.007 | 0.007 | 1.000 |
| *Eubacterium* | 0.09 | | 0.00 | 0.00 |  |  |  |
| *Z20* | 0.08 | | 0.00 | 0.00 | 0.007 | 0.007 | 1.000 |
| *Lachnospiraceae_ND3007_group* | 0.07 | | 0.00 | 0.00 | 0.025 | 0.025 | 1.000 |
| *uncultured_Ruminococcaceae_bacterium* | 0.04 | | 0.00 | 0.00 |  |  |  |
| *unclassified_Comamonadaceae* | 0.03 | | 0.00 | 0.00 | 0.075 | 0.075 | 1.000 |
| *Pygmaiobacter* | 0.03 | | 0.00 | 0.00 |  |  |  |
| *Olsenella* | 0.03 | | 0.00 | 0.00 | 0.024 | 0.024 | 1.000 |
