# Supplementary material for: Exploring the distinctive characteristics of gut microbiota across different horse breeds and ages using metataxonomics
Source: Front Cell Infect Microbiol. 2025 Jul 7;15:1590839. doi: 10.3389/fcimb.2025.1590839 (PMC12277257; doi:10.3389/fcimb.2025.1590839)
Supplement: Supplementary file 8 [file Table4.docx]

Supplementary Table S4 Differences in the relative abundance of phyla between ages (Wilcoxon rank-sum test, and *P*-values were corrected using the Benjamini-Hochberg method).

| Phylum | TBy (%) | TBo (%) | TBy vs TBo (*P*) |
| --- | --- | --- | --- |
| Bacillota | 48.58 | 53.24 | 0.175 |
| Bacteroidota | 27.22 | 30.37 | 0.347 |
| Pseudomonadota | 3.72 | 9.38 | 0.076 |
| Spirochaetota | 9.26 | 4.25 | 0.009 |
| Verrucomicrobiota | 4.87 | 1.81 | 0.047 |
| Actinobacteriota | 0.50 | 0.39 | 0.175 |
| Desulfobacterota | 0.23 | 0.19 | 0.403 |
| Patescibacteria | 0.51 | 0.14 | 0.009 |
| Fibrobacterota | 5.00 | 0.09 | 0.009 |
| Cyanobacteria | 0.07 | 0.07 | 0.917 |
| Synergistota | 0.00 | 0.04 | 0.037 |
| Deinococcota | 0.00 | 0.02 | 0.602 |
| Campylobacterota | 0.04 | 0.01 | 0.465 |
