# Supplementary material for: Exploring the distinctive characteristics of gut microbiota across different horse breeds and ages using metataxonomics
Source: Front Cell Infect Microbiol. 2025 Jul 7;15:1590839. doi: 10.3389/fcimb.2025.1590839 (PMC12277257; doi:10.3389/fcimb.2025.1590839)
Supplement: Supplementary file 9 [file Table5.docx]

Supplementary Table S5 Differences in the relative abundance of genera between ages (Wilcoxon rank-sum test, and *P*-values were corrected using the Benjamini-Hochberg method).

| Genus | TBy (%) | TBo (%) | TBy vs TBo (*P*) |
| --- | --- | --- | --- |
| Acinetobacter | 1.35 | 7.75 | 0.069 |
| Actinobacillus | 0.05 | 0.00 | 1.164 |
| Agathobacter | 0.14 | 0.00 | 0.582 |
| Akkermansia | 2.22 | 0.06 | 0.388 |
| Alloprevotella | 2.53 | 0.30 | 0.068 |
| Anaerofustis | 0.02 | 0.03 | 0.268 |
| Anaerovorax | 0.69 | 0.73 | 0.380 |
| Bacillus | 0.01 | 0.34 | 0.291 |
| Bacteroides | 0.17 | 0.00 | 0.233 |
| Bifidobacterium | 0.03 | 0.00 | 0.194 |
| Bradyrhizobium | 0.03 | 0.01 | 0.977 |
| Burkholderia_Caballeronia_Paraburkholderia | 0.02 | 0.00 | 0.725 |
| Butyrivibrio | 0.01 | 0.01 | 0.745 |
| Campylobacter | 0.04 | 0.01 | 0.588 |
| Candidatus_Saccharimonas | 0.51 | 0.10 | 0.166 |
| Candidatus_Soleaferrea | 0.18 | 0.16 | 0.824 |
| Caryophanon | 0.05 | 0.00 | 0.718 |
| Catenisphaera | 0.09 | 0.01 | 0.146 |
| Christensenellaceae_R_7_group | 1.27 | 2.72 | 0.129 |
| Clostridium_sensu_stricto_3 | 0.01 | 0.02 | 0.582 |
| Comamonas | 0.00 | 0.00 | 0.712 |
| Corynebacterium | 0.00 | 0.01 | 0.969 |
| Defluviitaleaceae_UCG_011 | 0.37 | 0.37 | 0.962 |
| Desulfovibrio | 0.23 | 0.19 | 0.520 |
| Erysipelatoclostridium | 0.04 | 0.03 | 0.467 |
| Escherichia_Shigella | 2.05 | 1.54 | 0.576 |
| FD2005 | 0.10 | 0.08 | 0.897 |
| Faecalibacterium | 0.21 | 0.00 | 0.116 |
| Family_XIII_AD3011_group | 0.36 | 0.10 | 0.106 |
| Fibrobacter | 5.00 | 0.09 | 0.097 |
| Galbibacter | 0.00 | 0.02 | 0.191 |
| Glutamicibacter | 0.00 | 0.03 | 0.429 |
| Incertae_Sedis | 0.14 | 0.05 | 0.090 |
| Kurthia | 0.14 | 0.00 | 0.189 |
| Lachnospiraceae_AC2044_group | 1.30 | 0.89 | 0.136 |
| Lachnospiraceae_ND3007_group | 0.00 | 0.03 | 0.187 |
| Lachnospiraceae_UCG_006 | 0.01 | 0.08 | 0.066 |
| Lachnospiraceae_UCG_008 | 0.11 | 0.24 | 0.045 |
| Lachnospiraceae_UCG_009 | 0.41 | 1.22 | 0.083 |
| Lachnospiraceae_XPB1014_group | 2.41 | 2.51 | 0.954 |
| Lactobacillus | 0.30 | 0.01 | 0.078 |
| Ligilactobacillus | 2.77 | 0.48 | 0.073 |
| Limosilactobacillus | 0.01 | 0.00 | 0.946 |
| Lysinibacillus | 3.27 | 4.31 | 0.425 |
| Membranicola | 0.00 | 0.01 | 0.420 |
| Mogibacterium | 0.02 | 0.02 | 1.000 |
| Monoglobus | 0.12 | 0.05 | 0.163 |
| NK4A214_group | 2.08 | 3.17 | 0.068 |
| Oribacterium | 0.38 | 0.87 | 0.065 |
| Oscillibacter | 0.02 | 0.00 | 0.415 |
| Pedobacter | 0.00 | 0.01 | 0.705 |
| Peptococcus | 0.00 | 0.06 | 0.061 |
| Phascolarctobacterium | 1.86 | 1.16 | 0.058 |
| Phoenicibacter | 0.00 | 0.16 | 0.055 |
| Prevotella | 1.02 | 0.11 | 0.053 |
| Prevotellaceae_UCG_001 | 3.17 | 6.38 | 0.092 |
| Prevotellaceae_UCG_003 | 0.33 | 0.23 | 0.065 |
| Prevotellaceae_UCG_004 | 1.62 | 1.72 | 0.939 |
| Pseudobutyrivibrio | 0.00 | 0.26 | 0.051 |
| Pseudomonas | 0.00 | 0.00 | 0.699 |
| Quinella | 0.27 | 0.09 | 0.044 |
| Rikenellaceae_RC9_gut_group | 6.88 | 9.93 | 0.049 |
| Rubellimicrobium | 0.00 | 0.00 | 0.693 |
| Ruminiclostridium | 0.18 | 0.51 | 0.047 |
| Ruminococcus | 0.10 | 2.98 | 0.045 |
| Rummeliibacillus | 0.00 | 0.81 | 0.043 |
| Saccharofermentans | 0.80 | 0.17 | 0.042 |
| Schwartzia | 0.00 | 0.00 | 0.931 |
| Shuttleworthia | 0.03 | 0.00 | 0.411 |
| Solibacillus | 13.19 | 11.08 | 0.924 |
| Sphaerochaeta | 0.31 | 0.21 | 0.184 |
| Streptococcus | 0.04 | 0.14 | 0.040 |
| Tannerella | 0.00 | 0.03 | 0.078 |
| Treponema | 8.91 | 3.97 | 0.039 |
| Truepera | 0.00 | 0.02 | 0.687 |
| UCG_002 | 1.31 | 0.80 | 0.161 |
| UCG_004 | 0.02 | 0.00 | 0.064 |
| UCG_005 | 1.62 | 1.02 | 0.038 |
| UCG_007 | 0.07 | 0.03 | 0.376 |
| UCG_009 | 0.07 | 0.00 | 0.036 |
| Weissella | 0.00 | 0.22 | 0.035 |
| Z20 | 0.00 | 0.05 | 0.076 |
| [Anaerorhabdus]_furcosa_group | 0.04 | 0.00 | 0.091 |
| [Eubacterium]_hallii_group | 0.01 | 0.06 | 0.090 |
| [Eubacterium]_ruminantium_group | 0.03 | 0.00 | 0.407 |
| [Eubacterium]_siraeum_group | 0.02 | 0.00 | 0.075 |
| hoa5_07d05_gut_group | 0.38 | 0.46 | 0.571 |
| unclassified_Anaerovoracaceae | 0.01 | 0.06 | 0.088 |
| unclassified_Bacteroidales | 0.70 | 0.11 | 0.034 |
| unclassified_Bacteroidales_BS11_gut_group | 0.17 | 0.51 | 0.043 |
| unclassified_Bacteroidales_RF16_group | 0.01 | 0.03 | 0.372 |
| unclassified_Bacteroidales_UCG_001 | 0.27 | 0.38 | 0.182 |
| unclassified_Bacteroidia | 0.09 | 0.20 | 0.462 |
| unclassified_Christensenellaceae | 0.29 | 0.80 | 0.033 |
| unclassified_Clostridia | 0.08 | 0.36 | 0.032 |
| unclassified_Clostridia_UCG_014 | 0.44 | 1.40 | 0.031 |
| unclassified_Coriobacteriales_Incertae_Sedis | 0.28 | 0.13 | 0.063 |
| unclassified_Dysgonomonadaceae | 0.01 | 0.13 | 0.031 |
| unclassified_Eggerthellaceae | 0.19 | 0.07 | 0.062 |
| unclassified_Enterobacteriaceae | 0.02 | 0.04 | 0.367 |
| unclassified_Erysipelotrichaceae | 0.25 | 0.31 | 0.159 |
| unclassified_F082 | 4.38 | 7.82 | 0.042 |
| unclassified_Fodinicurvataceae | 0.00 | 0.00 | 0.681 |
| unclassified_Gastranaerophilales | 0.04 | 0.02 | 0.515 |
| unclassified_Lachnospiraceae | 3.13 | 4.86 | 0.061 |
| unclassified_Marinifilaceae | 0.00 | 0.04 | 0.074 |
| unclassified_Muribaculaceae | 1.66 | 1.02 | 0.030 |
| unclassified_Oscillospiraceae | 0.15 | 0.32 | 0.029 |
| unclassified_Oscillospirales | 0.23 | 0.44 | 0.041 |
| unclassified_Oxalobacteraceae | 0.04 | 0.01 | 0.087 |
| unclassified_Peptococcaceae | 0.06 | 0.14 | 0.134 |
| unclassified_Planococcaceae | 2.35 | 1.15 | 0.028 |
| unclassified_Prevotellaceae | 0.12 | 0.00 | 0.028 |
| unclassified_RF39 | 0.00 | 0.01 | 0.402 |
| unclassified_Rhodospirillales | 0.07 | 0.00 | 0.027 |
| unclassified_Rikenellaceae | 0.14 | 0.04 | 0.026 |
| unclassified_Ruminococcaceae | 1.26 | 0.66 | 0.040 |
| unclassified_Selenomonadaceae | 0.00 | 0.06 | 0.026 |
| unclassified_Spirochaetaceae | 0.05 | 0.06 | 0.646 |
| unclassified_Succinivibrionaceae | 0.00 | 0.00 | 0.675 |
| unclassified_Synergistaceae | 0.00 | 0.04 | 0.073 |
| unclassified_Syntrophomonadaceae | 0.02 | 0.00 | 0.086 |
| unclassified_UCG_010 | 0.82 | 0.88 | 0.457 |
| unclassified_WCHB1_41 | 0.69 | 0.67 | 0.817 |
| unclassified_Xanthobacteraceae | 0.07 | 0.02 | 0.157 |
| unclassified_Xanthomonadaceae | 0.00 | 0.01 | 0.669 |
| unclassified_[Eubacterium]_coprostanoligenes_group | 1.60 | 2.65 | 0.132 |
| unclassified_p_251_o5 | 3.56 | 0.87 | 0.025 |
| uncultured_rumen_bacterium | 3.08 | 2.05 | 0.452 |
